# Moderate Exercise Has Limited but Distinguishable Effects on the Mouse Microbiome

**DOI:** 10.1128/mSystems.00006-17

**Published:** 2017-08-22

**Authors:** Emily V. Lamoureux, Scott A. Grandy, Morgan G. I. Langille

**Affiliations:** a Department of Pharmacology, Dalhousie University, Halifax, Nova Scotia, Canada; b School of Health and Human Performance (Kinesiology), Dalhousie University, Halifax, Nova Scotia, Canada; University of Colorado Denver

**Keywords:** 16S rRNA gene sequencing, exercise, gut microbiome, inflammation

## Abstract

The gut microbiome is known to have a complex yet vital relationship with host health. While both exercise and the gut microbiome have been shown to impact human health independently, the direct effects of moderate exercise on the intestinal microbiota remain unclear. In this study, we compared gut microbial diversity and changes in inflammatory markers associated with exercise over an 8-week period in mice that performed either voluntary exercise (VE) (*n* = 10) or moderate forced exercise (FE) (*n* = 11) and mice that did not perform any exercise (*n* = 21). VE mice, but not FE mice, had increased food intake and lean body mass compared to sedentary mice. The levels of inflammatory markers associated with exercise were similar for mice in all three groups. Traditional microbial profiles comparing operational taxonomic units (OTUs) in samples (*P* > 0.1) and multivariate analysis of beta diversity via Adonis testing (*P* > 0.1) did not identify significantly altered taxonomic profiles in the voluntary or forced exercise group compared to the sedentary controls. However, a random forests machine learning model, which takes into account the relationships between bacteria in a community, classified voluntary exercisers and nonexercisers with 97% accuracy at 8 weeks. The top bacteria used by the model allowed us to identify known taxa (*Bacteroides*, S24-7, and *Lactobacillus*) and novel taxa (*Rikenellaceae* and *Lachnospiraceae*) associated with exercise. Although aerobic exercise in mice did not result in significant changes of abundance in gut microbes or in host inflammatory response, more sophisticated computational methods could identify some microbial shifts. More study is needed on the effects of various exercise intensities and their impact on the gut microbiome.

**IMPORTANCE** The bacteria that live in our gut have a complex yet vital relationship with our health. Environmental factors that influence the gut microbiome are of great interest, as recent research demonstrates that these microbes, mostly bacteria, are important for normal host physiology. Diseases such as obesity, diabetes, inflammatory bowel disease, and colon cancer have also been linked to shifts in the microbiome. Exercise is known to have beneficial effects on these diseases; however, much less is known about its direct impact on the gut microbiome. Our results illustrate that exercise has a moderate but measurable effect on gut microbial communities in mice. These methods can be used to provide important insight into other factors affecting the microbiome and our health.

## Abstract (precis)

The bacteria that live in our gut have a complex yet vital relationship with our health. Environmental factors that influence the gut microbiome are of great interest, as recent research demonstrates that these microbes, mostly bacteria, are important for normal host physiology. Diseases such as obesity, diabetes, inflammatory bowel disease, and colon cancer have also been linked to shifts in the microbiome. Exercise is known to have beneficial effects on these diseases; however, much less is known about its direct impact on the gut microbiome. Our results illustrate that exercise has a moderate but measurable effect on gut microbial communities in mice. These methods can be used to provide important insight into other factors affecting the microbiome and our health.

## INTRODUCTION

The microbial populations that naturally inhabit the host are referred to as the microbiome. Changes to the host environment, such as selective pressures brought about by antibiotic use (1) or inflammation due to bacterial infection (2), can disrupt the community that is normally found in the host. In healthy individuals, the microbiotas are generally able to reestablish their functional niches after these types of disruptions (1–3). Conversely, it is also known that environmental factors, such as diet, contribute to changes in an individual’s gut microbiota throughout their lifetime (4).

Recent research into the human gut microbiome has revealed its complex relationship with human health. Intestinal microbiota has been shown to be an important contributor to normal host physiology, including immune development and the metabolism of energy and drugs (5, 6). Changes in the gut microbiota have been linked to obesity, diabetes, cardiovascular disease, inflammatory bowel disease, and colon cancer (7–13). Exercise has been shown to have beneficial effects on these same pathological states (14, 15), in part through the modulation of levels of inflammation (16). Furthermore, exercise has been shown to have both acute and chronic effects, and it is these chronic effects that have positive outcomes on disease states (17). However, what remains unclear is whether the chronic effects of exercise on inflammation alter the intestinal microbiome.

Several studies have attempted to describe the relationship between exercise and the microbiome. The impact of exercise with both age and nutrition has been studied in rodents (18–20). Mika et al. showed that age affects the impact that exercise has on the microbiome in rats (20). Young rats (3 weeks old) were more susceptible than adult rats (10 weeks old) to changes in microbial diversity as a result of exercise. Queipo-Ortuño et al. fed 6-week-old male rats either restrictively or *ad libitum*, with and without free access to an exercise wheel, and found that exercise increased gut bacterial diversity when the rats had unlimited access to food (18). This study was performed over only a 6-day period and limited by the use of PCR-DGGE (denaturing gradient gel electrophoresis), an insensitive method for microbial composition analysis. In contrast, a study examining the effects of both calorie restriction and voluntary exercise on the gut microbiome, also using 6-week-old male rats, found that exercise alone had no significant effects on microbial composition (19). The conclusions of this study were, however, largely focused on the diet component of the experiment, and fecal sampling did not start until 62 weeks into the study. A study by Cook et al. (21) demonstrated that voluntary wheel running attenuated, while forced treadmill running exacerbated disease progression in a mouse colitis model. When they compared the effects of voluntary and forced exercise on gut microbial diversity in healthy mice, they found both to have distinct and significant effects on community structure (22). In humans, elite athletes undergoing high-volume, high-intensity exercise had higher microbial diversity and metabolic pathways, as well as an increase in fecal metabolites such as short-chain fatty acids, but these differences could not be clearly separated from diet differences (23, 24).

In this study, we investigate the direct effects of exercise on the gut microbiome using both voluntary and moderate forced exercise models in mice, while controlling for diet and measuring changes in food intake, body mass composition, and host immunological expression.

## RESULTS

### Voluntary but not forced exercise results in higher food intake and lean mass.

**FIG 1:**
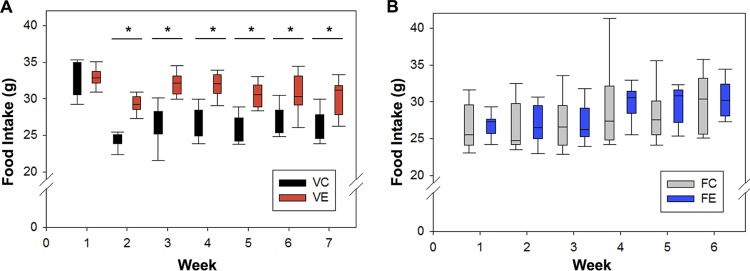
Voluntary but not forced exercise alters food consumption in mice. (A and B) Box plots depict average weekly food intake (in grams) of exercise and control mice for the voluntary exercise cohort (A) and the forced exercise cohort (B). Comparisons were conducted using two-sample *t* tests with a significance cutoff of *P* < 0.05. Values that are significantly different at time points are indicated with a bar and asterisk.

In the voluntary wheel running cohort, each voluntary exercise (VE) mouse had access to a running wheel at all times. The cumulative distance (in meters) traveled by each VE mouse was totalled for the 8 weeks. The mean total distance for the group was calculated at 138,565 m, with the mice running an average of 2.5 ± 0.7 km a day. In the forced treadmill running group, each exercise mouse ran for 40 min, 5 days a week. The cumulative distance (in meters) traveled by each forced exercise (FE) mouse was 21 km. The distances run per day by the FE mice were 600 m in weeks 1 and 2, 700 m in weeks 3 and 4, and 800 m in weeks 5 and 6. Food intake was measured per mouse on a weekly basis for the duration of the experiments (7 weeks for the voluntary cohort and 6 weeks for the forced cohort). A schematic of the experimental timeline is shown in Fig. S1 in the supplemental material. The cumulative food intake was averaged for each week (Fig. 1). Two-sample *t* tests for equal means were used to compare the mean cumulative food intake between exercise and control mice at each time point. Mice in the voluntary control (VC) group ate significantly less food overall than their VE counterparts, starting at week 2 (*P* < 0.05). Mice in the FE group had comparable food intake to the forced controls (FC) at each time point (*P* > 0.5).

10.1128/mSystems.00006-17.1 FIG S1  Experimental timeline for voluntary exercise and forced exercise cohorts. Voluntary wheel running took place over 8 weeks, while forced treadmill running was performed over 6 weeks. Fecal and weight samples were taken every 2 weeks, while blood and mucosal samples were taken at the experimental endpoint. DEXA scans for body mass composition were performed at week 6 of both studies. Download FIG S1, TIF file, 0.1 MB. Copyright © 2017 Lamoureux et al. 2017 Lamoureux et al. This content is distributed under the terms of the Creative Commons Attribution 4.0 International license.

**FIG 2:**
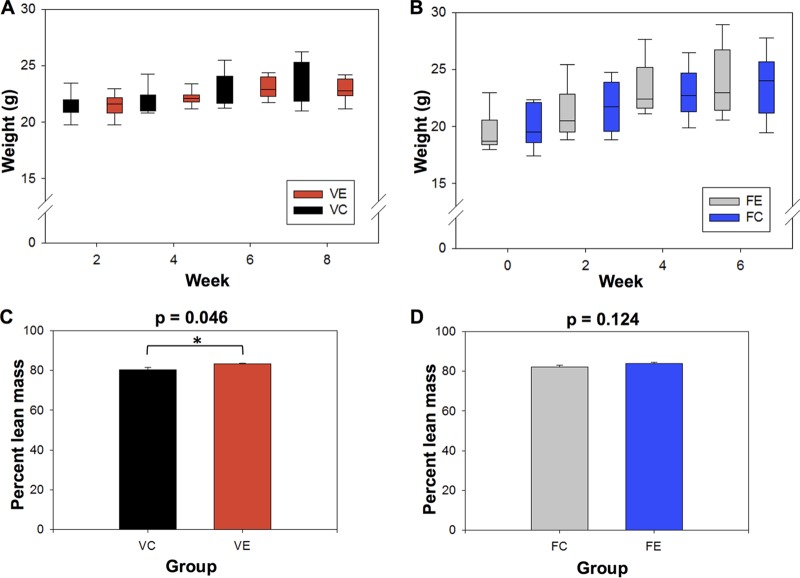
Voluntary but not forced exercise promotes lean body mass in mice. (A and B) Box plots depict average biweekly body mass measurements of exercise and control mice for the voluntary exercise cohort (A) and the forced exercise cohort (B). (C and D) Bar graphs illustrate percent lean body mass for the voluntary exercise cohort (*P* = 0.046) (C) and the forced exercise cohort (*P* = 0.124) (D). Comparisons were conducted using two-sample *t* tests with a significance cutoff of *P* < 0.05. Values that are significantly different at time points are indicated with a bar and asterisk.

Body mass measurements (in grams) for all mice were taken every 2 weeks (starting at week 2 for the voluntary cohort and week 0 for the forced cohort) of the experimental timeline (Fig. 2A and B). Two-sample *t* tests for equal means were used to compare the mean body weights for exercise and control mice at each time point. Body mass was not statistically different between the control and exercise mice in either cohort at any time point (*P* > 0.5). Dual-energy X-ray absorptiometry (DEXA) scans showed that lean body mass (calculated as a percentage of total body mass) (Fig. 2C and D) was significantly different between voluntary exercise mice and control mice (83.6% ± 1.2% for VE mice and 80.8% ± 0.5% for VC mice; *P* = 0.046) but did not differ between forced exercisers and controls (*P* > 0.1). When the forced exercise cohort was divided into male and female subsets, there was no significant difference between exercisers and controls.

### Voluntary and forced exercise have no measurable effect on bacterial diversity in the mouse microbiome.

**FIG 3:**
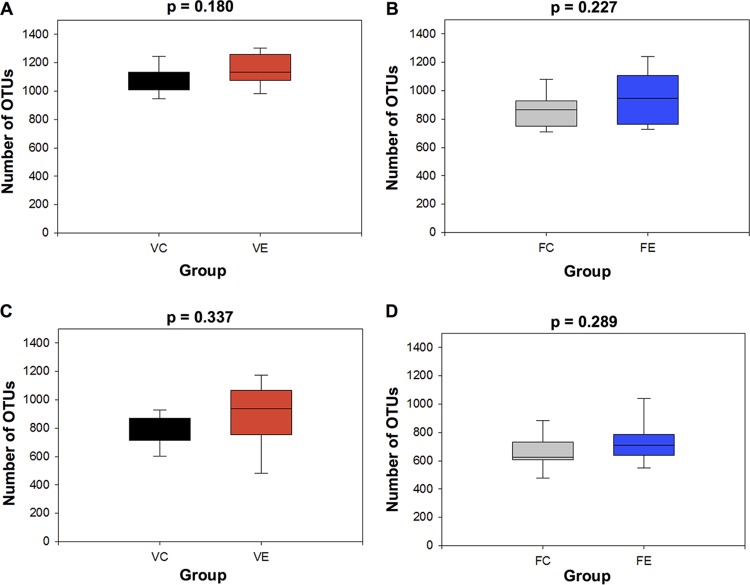
Voluntary and forced exercise do not affect species richness in the mouse gut. The number of OTUs from each fecal and mucosal sample at the final week (week 8 for VE and week 6 for FE) were counted, and counts per sample were averaged for each experimental group. Box plots illustrate average species richness of control and exercise groups for both voluntary exercise fecal samples (A) (*P* = 0.180) and mucosal samples (C) (*P* = 0.337) and forced exercise fecal samples (B) (*P* = 0.227) and mucosal samples (D) (*P* = 0.289). Comparisons were done using two-sample *t* tests.

**FIG 4:**
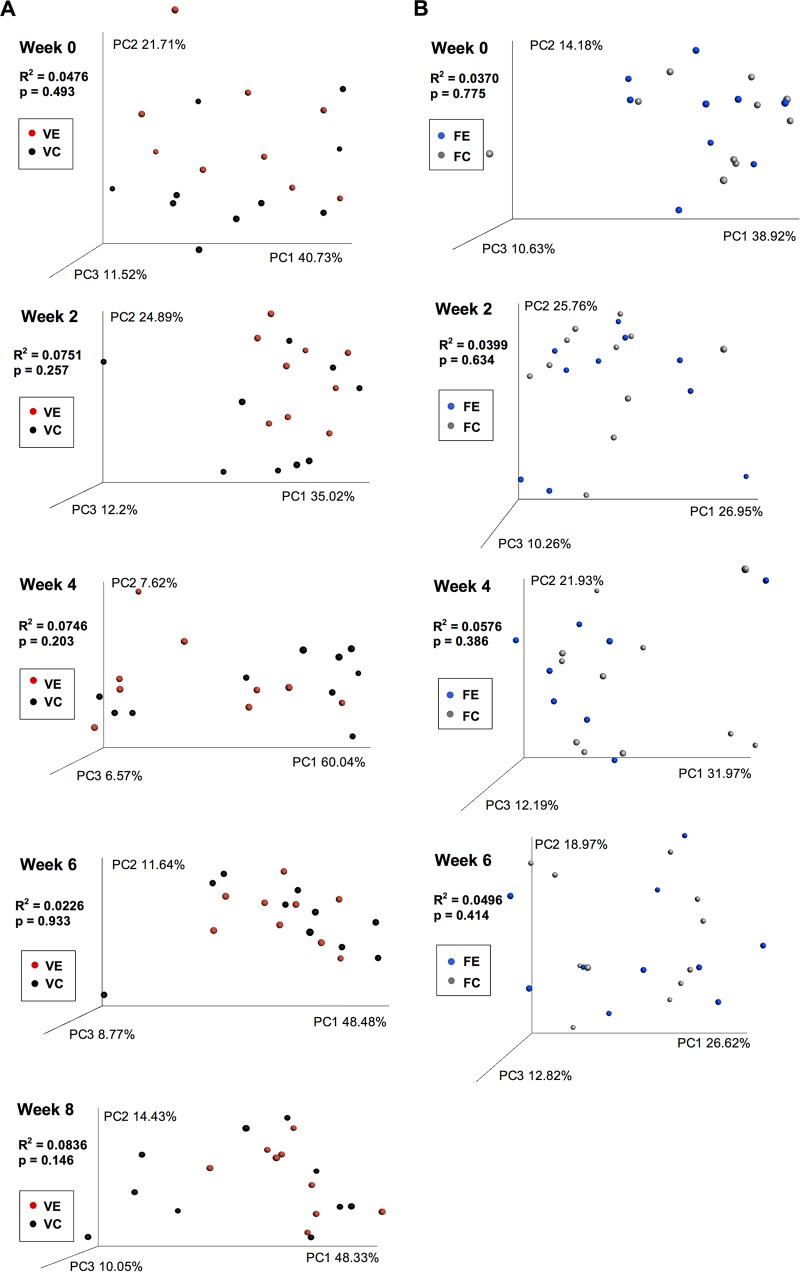
Gut microbial diversity of mice in voluntary and forced exercise groups. Weighted UniFrac principal coordinate axis plots compare the gut microbial diversity of exercise and control mice from the voluntary exercise cohort (A) and the forced exercise cohort (B). The beta diversity of fecal samples was compared using an Adonis test with a significance cutoff of *P* < 0.05. PC1, principal component 1.

Fecal samples were taken every 2 weeks from mice in both cohorts, while mucosal samples were taken at the experimental endpoint. There was no significant difference in alpha diversity (species richness) between exercise and control mice at week 8 in the voluntary exercise cohort (*P* = 0.180) or at week 6 in the forced exercise cohort (*P* = 0.227) (Fig. 3A and B). Species richness for mucosal samples was calculated in the same fashion (Fig. 3C and D) and was also not found to be different in either cohort (*P* = 0.337 for voluntary exercise; *P* = 0.289 for forced exercise). Taxonomic data from fecal samples were also used to generate weighted UniFrac beta-diversity principal coordinate axis plots for each time point using weighted (Fig. 4) and unweighted (Fig. S2) UniFrac beta-diversity measurements. An Adonis test did not reveal a significant difference in community structure between the fecal samples at each time point for the voluntary exercise cohort (week 0 [W0], *R*^2^ = 0.0476, *P* = 0.493; W2, *R*^2^ = 0.0751, *P* = 0.257; W4, *R*^2^ = 0.0746, *P* = 0.203; W6, *R*^2^ = 0.0226, *P* = 0.933; W8, *R*^2^ = 0.0836, *P* = 0.146) or forced exercise cohort (W0, *R*^2^ = 0.0370, *P* = 0.775; W2, *R*^2^ = 0.0399, *P* = 0.634; W4, *R*^2^ = 0.0576, *P* = 0.386; W6, *R*^2^ = 0.0496, *P* = 0.414) when using weighted UniFrac. Altering the beta-diversity measurement to unweighted UniFrac or Bray-Curtis (at different taxonomic levels) did not result in statistical significance (data not shown). Statistical comparisons of the relative abundances of individual taxa did not reveal any significant differences at any taxonomic level after multiple test correction.

10.1128/mSystems.00006-17.2 FIG S2  Gut microbial diversity of samples from mice in voluntary and forced exercise groups. Unweighted UniFrac principal coordinate axis plots compare gut microbial diversity of the exercising and control mice from the voluntary exercise cohort (A) (week 0 [W0], *R*^2^ = 0.0590, *P* = 0.289; W2, *R*^2^ = 0.0574, *P* = 0.467; W4, *R*^2^ = 0.0583, *P* = 0.289; W6, *R*^2^ = 0.0539, *P* = 480; W8, *R*^2^ = 0.0537, *P* = 0.512) and the forced exercise cohort (B) (W0, *R*^2^ = 0.0462, *P* = 0.955; W2, *R*^2^ = 0.0499, *P* = 0.437; W4, *R*^2^ = 0.0572, *P* = 0.363; W6, *R*^2^ = 0.0434, *P* = 0.800). The beta diversity of fecal samples was compared using an Adonis test with a significance cutoff of *P* < 0.05. Download FIG S2, TIF file, 0.1 MB. Copyright © 2017 Lamoureux et al. 2017 Lamoureux et al. This content is distributed under the terms of the Creative Commons Attribution 4.0 International license.

### Moderate exercise does not alter expression of inflammatory markers.

The results showed that serum levels of interleukin 1β (IL-1β), IL-6, and alpha tumor necrosis factor (TNF-α) did not differ significantly between the voluntary and forced exercise groups nor did the concentrations differ between either exercise group and the controls. The only marker found to have significantly different expression was keratinocyte-derived chemokine (KC), a neutrophil marker, homologous to IL-8 in humans (Table 1). KC was increased by almost 45% in voluntary exercisers alone (*P* < 0.001).

**TABLE 1:** Inflammatory cytokine profiles of control and exercising mice

Group	IL-1β concn (pg/ml)	IL-6 concn (pg/ml)	TNF-α concn (pg/ml)	KC concn (pg/ml)
Controls	212.79 ± 16.32	3.76 ± 0.40	155.68 ± 23.78	26.77 ± 1.45
Voluntary exercisers	227.10 ± 13.32	3.87 ± 0.45	173.02 ± 30.63	38.52 ± 1.52^a^
Forced exercisers	238.90 ± 11.07	4.96 ± 0.95	229.47 ± 60.37	27.85 ± 1.93

a Significantly different compared to the value for the control group.

### Machine learning identifies shifts in the mouse gut microbiome in response to exercise.

**FIG 5:**
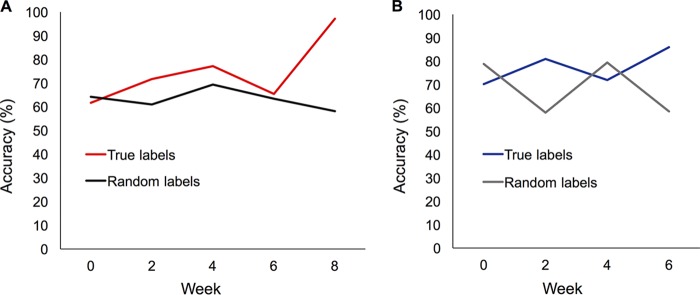
Accuracy of the random forests model in classifying exercise versus control samples. Sample OTU tables from exercise and control fecal samples for the voluntary exercise (A) and forced exercise (B) experiments was used to train a random forests classifier. Accuracy of the model using true category labels is plotted over time for both voluntary (97% at week 8) and forced (86% at week 6) cohorts. Accuracy using randomized category labels is also plotted over time for the voluntary exercise cohort (58% at week 8) and forced exercise cohort (58% at week 6).

Using Scikit-learn (25), a machine learning classification method known as random forests was trained and tested using a leave-one-out approach on the operational taxonomic unit (OTU) tables. In comparison to statistical comparisons of single taxa, machine learning can identify shifts in community structure that involve multiple taxa. Machine learning was able to distinguish between the microbiomes of VE and VC mice with 97% accuracy at week 8 and between FE and FC mice with 86% accuracy at week 6 (Fig. 5). Compared to a randomized model (where sample labels are randomized), the exercise microbiome could be accurately classified after 6 weeks of forced exercise and 8 weeks of voluntary exercise (Fig. 5).

The most important features/OTUs for classification as determined by the random forests model were inspected (Tables 2 and 3). We observed that in the voluntary exercise cohort, out of the top 30 taxa, 23 belong to the phylum *Bacteroidetes*, 4 belong to *Firmicutes*, 2 belong to *Proteobacteria*, and 1 belongs to *Actinobacteria*. Of the 23 taxa in the *Bacteroides* phylum, 18 of them are part of the S24-7 family, 4 are *Bacteroidaceae*, and 1 is *Rikenellaceae*. The four *Firmicutes* taxa fall into the order *Clostridiales* (Table 2). In the forced exercise cohort, 24 out of the top 30 taxa were from the *Firmicutes* phylum, while 6 were *Bacteroidetes*. Out of the 24 taxa in the *Firmicutes* phylum, 19 are in the order *Clostridiales*, 4 are in *Lactobacillales*, and 1 did not have an assigned order. Five out of the six taxa in the *Bacteroidetes* phylum belong to the *Bacteroides* genus, and the other is a *Parabacteroides* (Table 3).

**TABLE 2:**
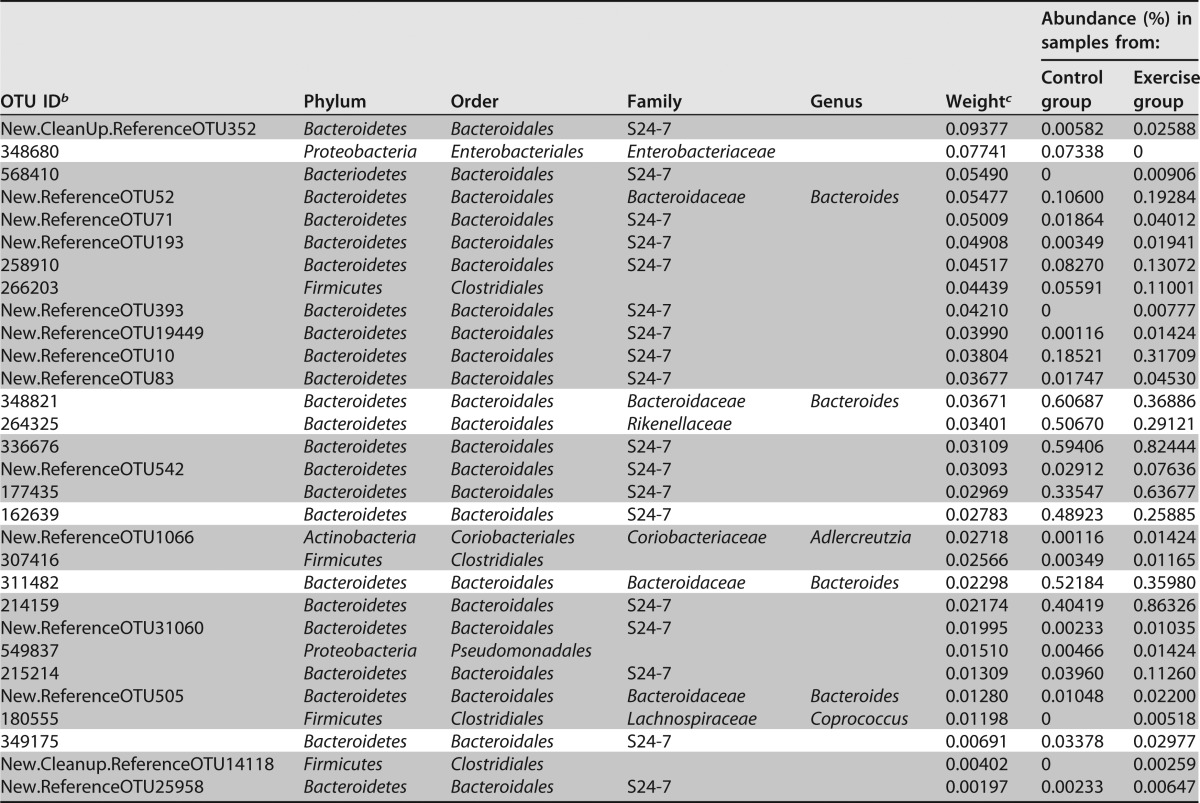
Top 30 OTUs important in classifying samples from mice in the control group versus voluntary exercise group^a^

a Taxa that increased with exercise are indicated by gray shading, and taxa that decreased with exercise are shown on white background.

^b^ ID, identification.

^c^ Weight refers to the importance that the random forests (RF) model accords to each taxon.

**TABLE 3:**
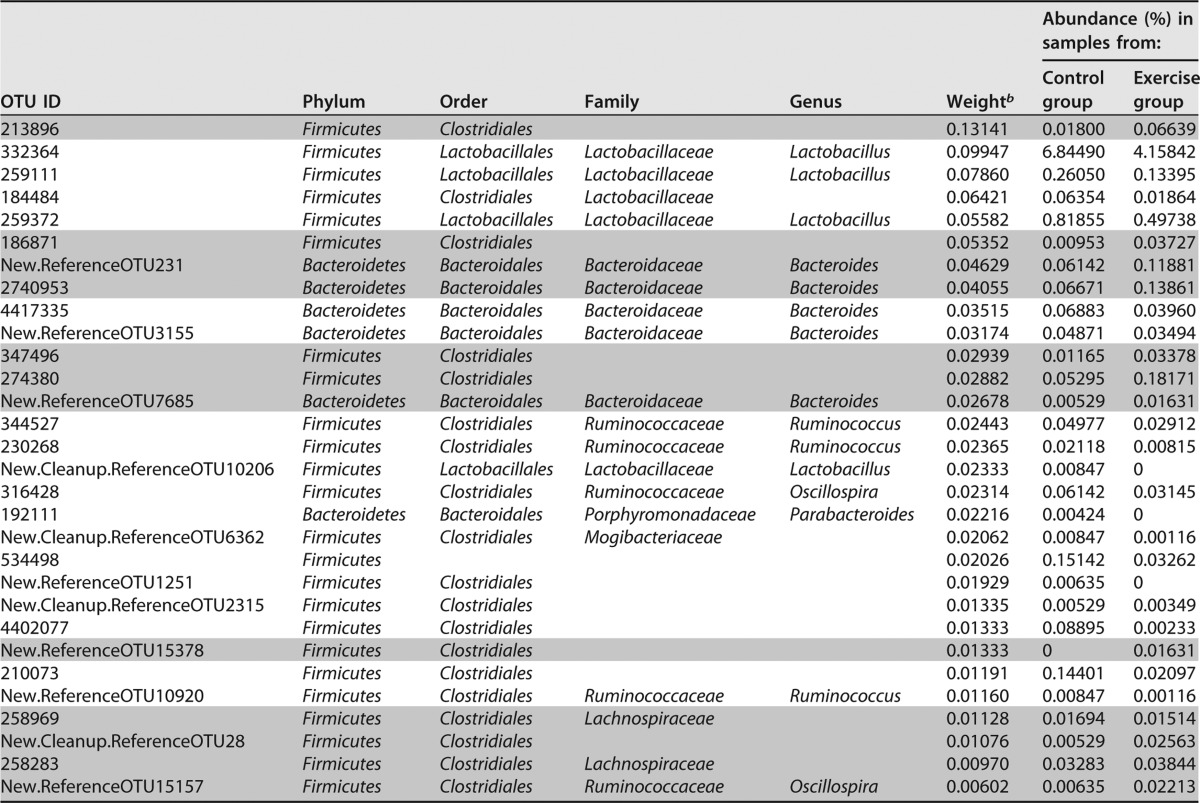
Top 30 OTUs important in classifying samples from the control group versus forced exercise group^a^

a Taxa that increased with exercise are indicated by gray shading, and taxa that decreased with exercise are shown on white background.

^b^ Weight refers to the importance that the random forests (RF) model accords to each taxon.

## DISCUSSION

The relationship between exercise and human health has been studied extensively, and it has been found that exercise makes a number of positive contributions to our health (26). Diet has also been shown to have a strong influence on our health and has been directly implicated in alteration of the gut microbiome (27). Environmental factors that influence gut flora are of great interest, as recent research demonstrates that these microbes are important for healthy host physiology. The effect that exercise has on gut microbial composition is an emerging field of interest, but so far, only a handful of studies have been done. Links between exercise, the gut microbiome, and disease have been studied through an increase in protective short-chain fatty acids (28, 29) and a decrease in bacteria associated with colorectal cancer and obesity (30, 31). These studies have not given consistent conclusions, so this study therefore aimed to characterize the impact that moderate exercise has on gut microbial diversity in a well-controlled setting. Controlled conditions included identical housing and food type, as well as regular measurements of food intake, body mass, and exercise levels.

Differences in body mass composition and food intake between control and exercise mice in the voluntary exercise study demonstrates that this exercise protocol did indeed have a tangible physiological effect on its subjects. Voluntary exercise mice not only had significantly less fat body mass and higher lean body mass than control mice, but they also maintained a higher level of food intake throughout the study. However, this effect was not seen in the forced exercise study, suggesting that this program did not induce the same stress in the mice as the voluntary one. This idea is supported in the inflammation data, as KC (IL-8 in humans), an exercise-induced cytokine, was higher in the voluntary cohort but not in the forced exercise cohort. Several studies have linked changes in inflammation to different forms of exercise in various populations (32–35). Some of this research has shown that both voluntary and forced exercise act to reduce proinflammatory cytokines. In our study, there was no change in either cohort in cytokines IL-1β, IL-6, and TNF-α, which are also commonly induced by strenuous exercise (36–38). This suggests that there was no chronic effect of exercise on serum levels of inflammatory markers.

Initial analyses of species richness and sample diversity show that neither exercise program appears to make a contribution to obvious microbiome changes. These observations contrast with previous studies that reported significant differences in the microbiome of both animal models (18, 19, 21, 22) and humans (23) in response to exercise. There are several reasons for these observational differences, which we attempted to address further. Initially, we used female mice for the voluntary wheel running cohort because it had been reported that female mice run more than male mice (39–41). During the course of our voluntary exercise study, Allen et al. found microbiome differences due to voluntary wheel running in male mice (22). Therefore, we decided to use both male and female mice in our forced exercise group; however, we did not find any sex differences between our mouse microbiomes, with or without exercise (data not shown). We also noted that Allen et al. (22) sequenced the V4 region of the 16S rRNA gene, while we had initially sequenced the V6-V8 region. Sequencing different hypervariable regions of the 16S rRNA gene has been shown to yield different results on the same data (42–44). Therefore, we conducted additional sequencing of the V4 region using the same primers as Allen et al. (22) but still found no significant differences in the microbiome in response to exercise. Last, we ran the same bioinformatic pipeline as described by Allen et al. with our data and found no significant differences, but when the data from their study were analyzed with the bioinformatics pipeline from this study, we replicated their published significant findings. It could be that other factors within previous studies including diet differences (19, 20), cage effects (18), age of mice (45), or slight differences in housing could be leading to contributing to some of the previously published observations. It is also very difficult to standardize the starting microbiota of animals in a study. Bar plots summarizing the taxa found in each of our treatment groups at the start of the study demonstrate different levels of major phyla between forced and voluntary exercise cohorts (see Fig. S3 in the supplemental material). Despite ordering the same strains of mice from the same breeding facility, environmental factors may cause microbiome shifts of significant magnitude to obscure treatment influences. Despite the lack of major observable differences in the microbiome, it was notable that more-complex methods that take into account microbial interactions like machine learning were able to distinguish subtle shifts in the mouse microbiome in response to exercise.

10.1128/mSystems.00006-17.3 FIG S3  Taxon summary of phyla in the starting microbiome of each treatment group. Bar plots show the average percentage of each phyla present in the gut of both control and exercising mice for the voluntary and forced cohorts at week 0 of the experimental timeline. Download FIG S3, TIF file, 0.1 MB. Copyright © 2017 Lamoureux et al. 2017 Lamoureux et al. This content is distributed under the terms of the Creative Commons Attribution 4.0 International license.

For both exercise cohorts, several known and novel associations with exercise were identified. The *Firmicutes* and *Bacteroidetes* phyla dominated the top 30 taxa important in differentiating between exercise and control treatments. Several other studies have linked these two phyla with response to exercise (18, 20, 23), along with the other two important phyla found in the voluntary exercise cohort, *Proteobacteria* and *Actinobacteria*. Within the *Bacteroidetes* phylum, at the genus level, *Bacteroides* remains an important taxon in both exercise cohorts, while the S24-7 family is relevant in the voluntary cohort. Out of 18 S24-7 OTUs, 16 increased with voluntary exercise. Within the *Firmicutes* phylum, taxa of the order *Clostridiales* are found to be important in both cohorts, while *Lactobacillus* is found to be a relevant genus in the forced exercise cohort, along with the *Ruminococcaceae* and *Mogibacteriaceae* families. All five of the *Lactobacillus* OTUs and all three of the *Ruminococcus* OTUs decreased with forced exercise. All of these taxa have previously been described in the literature in association with exercise (18, 20, 23). *Rikenellaceae*, a family in the *Clostridiales* order, was identified as a novel association within the voluntary exercise cohort, while *Lachnospiraceae*, a family in the *Bacteroidales* order was a novel association found in both cohorts. Several studies have also described changes in the levels of *Bifidobacterium*, *Prevotella*, and *Erysipelotrichaceae* species as a result of exercise in mice and humans (18, 20, 22, 23).

The higher classification accuracy results within the voluntary versus forced exercise mice again support that the voluntary exercise model was more vigorous overall. Out of the important taxa shared across both exercise programs, it is known that taxa in the *Bacteroidales* and *Clostridiales* orders produce short-chain fatty acids (46) that protect against obesity and colon cancer (47, 48). *Lactobacillus* species, found to be important only in the forced exercise cohort, also produce short-chain fatty acids such as butyrate and have been shown to be protective against pathogens (18). *Lactobacillus* species are also implicated in fat storage; some increase fat storage, while others decrease it (49–51). The bacterial families *Ruminococceae* and *Mogibacteriaceae*, also identified in the forced exercise cohort are associated with leanness (52). Out of the important taxa which are novel associations with exercise, bacteria of the *Lachnospiraceae* and *Rikenellaceae* families have beneficial effects as mentioned above (53, 54).

### Conclusions.

Contrary to previous studies (21, 22), our initial observations of bacterial diversity indicate limited alterations to the microbiome in response to moderate exercise. Inflammatory profiles were also not found to be altered from this exercise. However, a supervised random forest trained model was able to classify mice as sedentary or exercising based on their microbiome with 97% accuracy for voluntary exercise modality and 86% for forced exercise modality. Compared to other known environmental drivers such as diet, moderate exercise may play a more limited role in shaping the gut microbiome. Our results are from healthy, young, nonobese mice, and more study is needed to understand the dynamics and interplay between exercise and these other important factors on the human microbiome.

## MATERIALS AND METHODS

Forty-two 6- to 10-week-old C57BL/6 mice (11 male, 31 female) were obtained from Charles River Laboratory (Canada). All mice were housed individually on a 12-h light/12-h dark cycle in the University Animal Care facility for the duration of the experiment. Mice were assigned to either a voluntary exercise (VE) group (*n* = 10), a forced exercise (FE) group (*n* = 11), or a nonexercise control group (*n* = 21). All protocols were conducted in accordance with the Canadian Council on Animal Care guidelines and approved by the Dalhousie University Committee on Laboratory Animals.

### Experimental conditions. (i) Voluntary wheel running.

Twenty female mice were used for the 8-week voluntary exercise portion of the study. Upon arrival, mice were allowed to acclimatize in individual housing for 1 week and then were randomly assigned to a voluntary wheel running (VE) group (*n* = 10) or a sedentary control (VC) group (*n* = 10). Mice in the VE group were housed in cages that contained running wheels, giving the mice 24-h access to the running wheel. Each running wheel was connected to a data-logger, which counted the number of wheel revolutions per day for the 55-day study period. Using the diameter of the wheel, the total number of running wheel revolutions were converted to meters traveled per day.

### (ii) Forced treadmill running.

Eleven mice (five female, six male) were randomly assigned to a forced treadmill running (FE) group. Mice were allowed to acclimatize in individual housing for 1 week, and then FE mice were exposed to the treadmill for 5 days prior to starting the training protocol. Following acclimation to the treadmill, a forced running protocol was administered for 6 weeks. Five days a week, FE mice were run for 40 min, starting at a speed of 15 m/min for weeks 1 and 2, and increasing by 2.5 m/min every 2 weeks, so that by weeks 5 and 6, the mice were running at a speed of 20 m/min. Benito et al. (55) have previously defined 60 min of treadmill running at 36 m/min as vigorous exercise for rats, and we therefore defined our forced treadmill running protocol (40 min at 15 to 20 m/min) as moderate exercise in mice. Control (FC) mice were placed in a clean, empty cage for a comparable amount of time to mimic handling stress. Mice were trained on the LE8700 single-lane treadmill equipped with a rest platform and an electronic control unit (Panlab Harvard Apparatus). When mice stopped running, they were gently nudged off the rest platform and back onto the treadmill belt.

### Diet and body mass composition.

All exercising and control animals had free access to the same food (Prolab RMH 3000; LabDiet, Brentwood, MO) and water. Food was weighed out and distributed to each mouse’s cage every 7 days, and uneaten food from the previous 7 days was weighed to determine how much food had been consumed.

To assess body composition, mice were weighed every 2 weeks. Dual-energy X-ray absorptiometry (DEXA) was also used to assess body mass composition of the mice. In brief, mice were anesthetized with isofluorane and placed in a prostrate position. They were scanned using a Lunar PIXImus2 (GE Medical Systems) DEXA machine. Whole-body scans, minus the head, were taken and bone marrow density (in grams per square centimeter), bone marrow content (in grams), body area (in square centimeters), lean mass (in grams), and fat mass (in grams) were determined. DEXA scans were taken at week 6 of both exercise protocols.

### Fecal sample collection.

Starting on day zero of the experimental timeline, fecal samples were collected on a biweekly basis by placing each mouse in a separate clean cage, waiting until they passed fecal pellets, and then transferring these pellets to autoclaved microcentrifuge tubes using sterile forceps. Fecal samples were stored at −80°C until they were required for analysis.

### Terminal sample collection.

At the end time point of both experimental protocols, the mice were sacrificed by cervical dislocation while under isofluorane anesthesia. The chest cavity was then rapidly opened, the aorta was cut, and blood was collected from the chest cavity and placed in a 2-ml Eppendorf tube. The blood samples were allowed to sit at room temperature for 15 min and then spun at 10,000 × *g* for 15 min at 4°C. The serum was then removed and stored at −80°C until required for analysis.

The terminal half of the colon was removed from the animals, and any fecal contents were flushed out with cold phosphate-buffered saline (PBS) using a rat feeding tube. An incision was then made longitudinally along the colon, and mucosal contents were scraped off using a glass coverslip and deposited into an Eppendorf tube. All fecal and mucosal samples were frozen immediately using liquid nitrogen and stored at −80°C.

### DNA isolation, library preparation, and sequencing.

DNA was isolated from fecal and mucosal samples using the PowerFecal DNA isolation kit (Mo Bio Laboratories). Briefly, the protocol follows. In a tube containing garnet beads and lysis buffer, samples are heated and then homogenized by bead-beating (disruptor-Genie). After centrifugation, non-DNA organic and inorganic cell contents are precipitated from the supernatant. A high-concentration salt solution is then added to the supernatant to allow DNA to selectively bind the silica membrane of a spin filter column. After being bound to the column and washed, purified DNA is eluted in low-salt conditions.

Variable regions V6-V8 of bacterial 16S rRNA genes were amplified from all purified DNA using the PCR conditions and primers from Comeau et al. (56), modified for use on the Illumina MiSeq. The forward and reverse primers used Nextera Illumina index tags and sequencing adapters fused to the 16S rRNA gene-specific sequences. Each sample was amplified with a different combination of index tags to allow for sample identification after multiplex sequencing. Following 16S rRNA gene amplification, paired-end 300-bp plus 300-bp V3 sequencing was performed for all samples on the Illumina MiSeq.

### Bioinformatic analysis.

Analysis of sequencing data was done on a Linux virtual machine, using the Microbiome Helper workflow, specific to 16S rRNA gene analysis, obtained from GitHub (https://github.com/mlangill/microbiome_helper/wiki/16S-standard-operating-procedure). Paired-end reads were stitched together using PEAR (57), and then low-quality reads that are less than 400 bp long and have less than 90% of their bases at a quality score of 30 or more were filtered. Chimeras were removed using VSEARCH (https://github.com/torognes/vsearch). Operational taxonomic units (OTUs) were generated within QIIME (58) through the open-reference OTU picking protocol (59) at 97% identity against the GreenGenes database v13_5 (60). Open-reference picking assigns OTUs by first mapping sequence reads first to a reference genome database using SortMeRNA (61). Any sequences that fail to align with known sequences are aligned *de novo*, meaning that they are clustered with each other based on similarity using SumaClust (https://git.metabarcoding.org/obitools/sumaclust/wikis/home). OTUs with low counts (based on a dynamic cutoff of 0.1% of the total number of sequences per sample) were removed, which has been previously shown to ensure that the number of OTUs is accurately represented (62). Postfiltering, the average sequence coverage for fecal and mucosal samples, respectively, was 23,558 and 28,865 sequences/sample. For comparison of microbial communities across experimental groups, fecal and mucosal samples were normalized to a depth of 8,585 and 7,081 reads/sample, respectively. Four fecal samples and two mucosal samples were excluded due to low coverage.

UniFrac beta-diversity plots were generated using information from the OTU table to illustrate microbial diversity between exercise and control samples across all time points (63). Beta-diversity plots used principal coordinate analysis (PCoA) to illustrate the variation in the data. Linear equations were fitted to the data, so that each equation explained the most amount of variation possible (principal components). The three largest principal components were then assigned to the *x*, *y*, and *z* axes of a three-dimensional plot. Each sample is assigned a value based on its principal components and plotted, with relative proximity to other samples in three-dimensional space correlating to sample similarity.

### Inflammatory profile analysis.

Inflammatory marker concentrations in serum (IL-1β, IL-16, IL-10, and TNF-α) were measured in blood samples collected 2 days after the exercise endpoint using a custom mouse multiplex assay (Bio-Rad). The assay was prepared according to the manufacturer’s instructions and read using a MagPix multiplex reader (Bio-Rad). Initial serum samples were diluted by a factor of 4. All samples were run in singlicate. A one-way analysis of variance (ANOVA) was used to determine whether any differences existed between groups for the aforementioned cytokines.

### Statistical analyses for microbial samples.

For all analyses, the *P* value cutoff is 0.05, and standard errors are reported.

An Adonis test as implemented within the compare_categories.py QIIME script (58) was used to compare treatment groups and determine whether exercise significantly affects gut microbial diversity. Adonis is a nonparametric multivariate analysis of variance which in this case compares the abundance of each bacteria in a sample to its abundance in other samples. It tests the null hypothesis that the bacterial composition of the samples is the same in control and exercise groups. The *R*^2^ value is the effect size and indicates the percent variation that can be explained by the tested variable, in this case exercise. The graphical software package STAMP (64) was also used to determine whether exercise significantly affected levels of individual taxa using unpaired *t* test with Benjamini-Hochberg false-discovery rate (FDR) and a *P* value cutoff of 0.05.

### Machine learning.

In order to examine subtle changes in community structure, we employed a form of machine learning, known as supervised learning, where features (in this case OTUs present in the samples) are used to predict the class (experimental condition) to which a sample belongs. Fecal OTU data from the two exercise cohorts was input into the python software Scikit-learn (25) to build separate random forests (RF) models. Using a leave-one-out method, the models were trained to classify samples from both cohorts as either exercise or control based on their OTU profile. A parameter search from 1 to 30 trees was tested to determine the highest accuracy. Accuracy was reported as the mean of 100 iterations of modeling and testing. Importance of features as output by random forests were averaged across the iterations and were used to determine the taxa most important for classification.

The random forests model was run with the OTU data 100 times, for both the voluntary and forced cohorts. The average weights for each OTU were computed, and the top 30 OTUs for each cohort were selected. The model was run again 100 times using only the selected OTUs, and the average classification accuracy for samples at each time point was calculated using both true and randomized sample labels.

### Accession number(s).

The raw 16S rRNA gene data supporting the conclusions of this article are available in the European Nucleotide Archive under accession number PRJEB18615.

## References

[1] Raymond F, Ouameur AA, Déraspe M, Iqbal N, Gingras H, Dridi B, Leprohon P, Plante PL, Giroux R, Bérubé È, Frenette J, Boudreau DK, Simard JL, Chabot I, Domingo MC, Trottier S, Boissinot M, Huletsky A, Roy PH, Ouellette M, Bergeron MG, Corbeil J 2016 The initial state of the human gut microbiome determines its reshaping by antibiotics. ISME J 10:707–720. doi:10.1038/ismej.2015.148. 26359913 PMC4817689

[2] Sekirov I, Russell SL, Antunes LC, Finlay BB 2010 Gut microbiota in health and disease. Physiol Rev 90:859–904. doi:10.1152/physrev.00045.2009. 20664075

[3] Dethlefsen L, Huse S, Sogin ML, Relman DA 2008 The pervasive effects of an antibiotic on the human gut microbiota, as revealed by deep 16S rRNA sequencing. PLoS Biol 6:e280. doi:10.1371/journal.pbio.0060280. 19018661 PMC2586385

[4] David LA, Maurice CF, Carmody RN, Gootenberg DB, Button JE, Wolfe BE, Ling AV, Devlin AS, Varma Y, Fischbach MA, Biddinger SB, Dutton RJ, Turnbaugh PJ 2013 Diet rapidly and reproducibly alters the human gut microbiome. Nature 505:559–563. doi:10.1038/nature12820. 24336217 PMC3957428

[5] Kinross JM, Darzi AW, Nicholson JK 2011 Gut microbiome-host interactions in health and disease. Genome Med 3:14. doi:10.1186/gm228. 21392406 PMC3092099

[6] Cani PD, Delzenne NM 2009 The role of the gut microbiota in energy metabolism and metabolic disease. Curr Pharm Des 15:1546–1558. doi:10.2174/138161209788168164. 19442172

[7] Ettinger G, MacDonald K, Reid G, Burton JP 2014 The influence of the human microbiome and probiotics on cardiovascular health. Gut Microbes 5:719–728. doi:10.4161/19490976.2014.983775. 25529048 PMC4615746

[8] Serino M, Blasco-Baque V, Nicolas S, Burcelin R 2014 Far from the eyes, close to the heart: dysbiosis of gut microbiota and cardiovascular consequences. Curr Cardiol Rep 16:540. doi:10.1007/s11886-014-0540-1. 25303894 PMC4194023

[9] Everard A, Belzer C, Geurts L, Ouwerkerk JP, Druart C, Bindels LB, Guiot Y, Derrien M, Muccioli GG, Delzenne NM, de Vos WM, Cani PD 2013 Cross-talk between Akkermansia muciniphila and intestinal epithelium controls diet-induced obesity. Proc Natl Acad Sci U S A 110:9066–9071. doi:10.1073/pnas.1219451110. 23671105 PMC3670398

[10] Qin J, Li Y, Cai Z, Li S, Zhu J, Zhang F, Liang S, Zhang W, Guan Y, Shen D, Peng Y, Zhang D, Jie Z, Wu W, Qin Y, Xue W, Li J, Han L, Lu D, Wu P, Dai Y, Sun X, Li Z, Tang A, Zhong S, Li X, Chen W, Xu R, Wang M, Feng Q, Gong M, Yu J, Zhang Y, Zhang M, Hansen T, Sanchez G, Raes J, Falony G, Okuda S, Almeida M, LeChatelier E, Renault P, Pons N, Batto J-M, Zhang Z, Chen H, Yang R, Zheng W, Li S, Yang H, Wang J, Ehrlich SD, Nielsen R, Pedersen O, Kristiansen K, Wang J 2012 A metagenome-wide association study of gut microbiota in type 2 diabetes. Nature 490:55–60. doi:10.1038/nature11450. 23023125

[11] Sha S, Xin B, Wang X, Zhang Y, Wang H, Kong X, Zhu H, Wu K 2012 The biodiversity and composition of the dominant fecal microbiota of patients with inflammatory bowel disease. Diagn Microbiol Infect Dis 75:245–251. doi:10.1016/j.diagmicrobio.2012.11.022. 23276768

[12] Scanlan PD, Shanahan F, Clune Y, Collins JK, O’Sullivan GC, O’Riordan M, Holmes E, Wang Y, Marchesi JR 2008 Culture-independent analysis of the gut microbiota in colorectal cancer and polyposis. Environ Microbiol 10:789–798. doi:10.1111/j.1462-2920.2007.01503.x. 18237311

[13] Turnbaugh PJ, Ley RE, Mahowald MA, Magrini V, Mardis ER, Gordon JI 2006 An obesity-associated gut microbiome with increased capacity for energy harvest. Nature 444:1027–1031. doi:10.1038/nature05414. 17183312

[14] Hägg U, Wandt B, Bergström G, Volkmann R, Gan LM 2005 Physical exercise capacity is associated with coronary and peripheral vascular function in healthy young adults. Am J Physiol Heart Circ Physiol 289:H1627–H1634. doi:10.1152/ajpheart.00135.2005. 15937100

[15] Peters HP, De Vries WR, Vanberge-Henegouwen GP, Akkermans LM 2001 Potential benefits and hazards of physical activity and exercise on the gastrointestinal tract. Gut 48:435–439. doi:10.1136/gut.48.3.435. 11171839 PMC1760153

[16] Pedersen BK, Hoffman-Goetz L 2000 Exercise and the immune system: regulation, integration and adaptation. Physiol Rev 80:1055–1081. 10893431 10.1152/physrev.2000.80.3.1055

[17] Chen YW, Apostolakis S, Lip GYH 2014 Exercise-induced changes in inflammatory processes: implications for thrombogenesis in cardiovascular disease. Ann Med 46:439–455. doi:10.3109/07853890.2014.927713. 25012964

[18] Queipo-Ortuño MI, Seoane LM, Murri M, Pardo M, Gomez-Zumaquero JM, Cardona F, Casanueva F, Tinahones FJ 2013 Gut microbiota composition in male rat models under different nutritional status and physical activity and its association with serum leptin and ghrelin levels. PLoS One 8:e65465. doi:10.1371/journal.pone.0065465. 23724144 PMC3665787

[19] Zhang C, Li S, Yang L, Huang P, Li W, Wang S, Zhao G, Zhang M, Pang X, Yan Z, Liu Y, Zhao L 2013 Structural modulation of gut microbiota in life-long calorie-restricted mice. Nat Commun 4:2163. doi:10.1038/ncomms3163. 23860099 PMC3717500

[20] Mika A, Van Treuren W, González A, Herrera JJ, Knight R, Fleshner M 2015 Exercise is more effective at altering gut microbial composition and producing stable changes in lean mass in juvenile versus adult male F344 rats. PLoS One 10:e0125889. doi:10.1371/journal.pone.0125889. 26016739 PMC4446322

[21] Cook MD, Martin SA, Williams C, Whitlock K, Wallig MA, Pence BD, Woods JA 2013 Forced treadmill exercise training exacerbates inflammation and causes mortality while voluntary wheel training is protective in a mouse model of colitis. Brain Behav Immun 33:46–56. doi:10.1016/j.bbi.2013.05.005. 23707215 PMC3775960

[22] Allen JM, Berg Miller MEB, Pence BD, Whitlock K, Nehra V, Gaskins HR, White BA, Fryer JD, Woods JA 2015 Voluntary and forced exercise differentially alters the gut microbiome in C57BL/6J mice. J Appl Physiol 118:1059–1066. doi:10.1152/japplphysiol.01077.2014. 25678701

[23] Clarke SF, Murphy EF, O’Sullivan O, Lucey AJ, Humphreys M, Hogan A, Hayes P, O’Reilly M, Jeffery IB, Wood-Martin R, Kerins DM, Quigley E, Ross RP, O’Toole PW, Molloy MG, Falvey E, Shanahan F, Cotter PD 2014 Exercise and associated dietary extremes impact on gut microbial diversity. Gut 63:1913–1920. doi:10.1136/gutjnl-2013-306541. 25021423

[24] Barton W, Penney NC, Cronin O, Garcia-Perez I, Molloy MG, Holmes E, Shanahan F, Cotter PD, O’Sullivan O 30 3 2017 The microbiome of professional athletes differs from that of more sedentary subjects in composition and particularly at the functional metabolic level. Gut doi:10.1136/gutjnl-2016-313627. 28360096

[25] Pedregosa F, Varoquaux G, Gramfort A, Michel V, Thirion B, Grisel O, Blondel M, Prettenhofer P, Weiss R, Dubourg V, Vanderplas J, Passos A, Cournapeau D, Brucher M, Perrot M, Duchesnay É 2011 Scikit-learn: machine learning in Python. J Mach Learn Res 12:2825–2830.

[26] Dangardt FJ, McKenna WJ, Lüscher TF, Deanfield JE 2013 Exercise: friend or foe? Nat Rev Cardiol 10:495–507. doi:10.1038/nrcardio.2013.90. 23797794

[27] Sonnenburg JL, Bäckhed F 2016 Diet-microbiota interactions as moderators of human metabolism. Nature 535:56–64. doi:10.1038/nature18846. 27383980 PMC5991619

[28] Matsumoto M, Inoue R, Tsukahara T, Ushida K, Chiji H, Matsubara N, Hara H 2008 Voluntary running exercise alters microbiota composition and increases n-butyrate concentration in the rat cecum. Biosci Biotechnol Biochem 72:572–576. doi:10.1271/bbb.70474. 18256465

[29] Perrin P, Pierre F, Patry Y, Champ M, Berreur M, Pradal G, Bornet F, Meflah K, Menanteau J 2001 Only fibres promoting a stable butyrate producing colonic ecosystem decrease the rate of aberrant crypt foci in rats. Gut 48:53–61. doi:10.1136/gut.48.1.53. 11115823 PMC1728184

[30] Choi JJ, Eum SY, Rampersaud E, Daunert S, Abreu MT, Toborek M 2013 Exercise attenuates PCB-induced changes in the mouse gut microbiome. Environ Health Perspect 121:725–730. doi:10.1289/ehp.1306534. 23632211 PMC3672930

[31] Turnbaugh PJ, Bäckhed F, Fulton L, Gordon JI 2008 Diet-induced obesity is linked to marked but reversible alterations in the mouse distal gut microbiome. Cell Host Microbe 3:213–223. doi:10.1016/j.chom.2008.02.015. 18407065 PMC3687783

[32] Allen J, Sun Y, Woods JA 2015 Exercise and the regulation of inflammatory responses. Prog Mol Biol Transl Sci 135:337–354. doi:10.1016/bs.pmbts.2015.07.003. 26477921

[33] Bilski J, Mazur-Bialy A, Brzozowski B, Magierowski M, Zahradnik-Bilska J, Wójcik D, Magierowska K, Kwiecien S, Mach T, Brzozowski T 2016 Can exercise affect the course of inflammatory bowel disease? Experimental and clinical evidence. Pharmacol Rep 68:827–836. doi:10.1016/j.pharep.2016.04.009. 27255494

[34] Cook MD, Allen JM, Pence BD, Wallig MA, Gaskins HR, White BA, Woods JA 2016 Exercise and gut immune function: evidence of alterations in colon immune cell homeostasis and microbiome characteristics with exercise training. Immunol Cell Biol 94:158–163. doi:10.1038/icb.2015.108. 26626721

[35] Crimi E, Ignarro LJ, Cacciatore F, Napoli C 2009 Mechanisms by which exercise training benefits patients with heart failure. Nat Rev Cardiol 6:292–300. doi:10.1038/nrcardio.2009.8. 19352333

[36] Donovan DC, Jackson CA, Colahan PT, Norton N, Hurley DJ 2007 Exercise-induced alterations in pro-inflammatory cytokines and prostaglandin F2α in horses. Vet Immunol Immunopathol 118:263–269. doi:10.1016/j.vetimm.2007.05.015. 17617470

[37] Nielsen AR, Pedersen BK 2007 The biological roles of exercise-induced cytokines: IL-6, IL-8, and IL-15. Appl Physiol Nutr Metab 32:833–839. doi:10.1139/H07-054. 18059606

[38] Pedersen BK 2000 Exercise and cytokines. Immunol Cell Biol 78:532–535. doi:10.1111/j.1440-1711.2000.t01-11-.x. 11050536

[39] Bartling B, Al-Robaiy S, Lehnich H, Binder L, Hiebl B, Simm A 2017 Sex-related differences in the wheel-running activity of mice decline with increasing age. Exp Gerontol 87:139–147. doi:10.1016/j.exger.2016.04.011. 27108181

[40] Konhilas JP, Maass AH, Luckey SW, Stauffer BL, Olson EN, Leinwand LA 2004 Sex modifies exercise and cardiac adaptation in mice. Am J Physiol Heart Circ Physiol 287:H2768–H2776. doi:10.1152/ajpheart.00292.2004. 15319208 PMC2637113

[41] McMullan RC, Kelly SA, Hua K, Buckley BK, Faber JE, Pardo-Manuel de Villena F, Pomp D 2016 Long-term exercise in mice has sex-dependent benefits on body composition and metabolism during aging. Physiol Rep 4:e13011. doi:10.14814/phy2.13011. 27905293 PMC5112492

[42] Huse SM, Dethlefsen L, Huber JA, Welch DM, Relman DA, Sogin ML 2008 Exploring microbial diversity and taxonomy using SSU rRNA hypervariable tag sequencing. PLOS Genet 4:e1000255. doi:10.1371/journal.pgen.1000255. 19023400 PMC2577301

[43] Kim M, Morrison M, Yu Z 2011 Evaluation of different partial 16S rRNA gene sequence regions for phylogenetic analysis of microbiomes. J Microbiol Methods 84:81–87. doi:10.1016/j.mimet.2010.10.020. 21047533

[44] Kumar PS, Brooker MR, Dowd SE, Camerlengo T 2011 Target region selection is a critical determinant of community fingerprints generated by 16S pyrosequencing. PLoS One 6:e20956. doi:10.1371/journal.pone.0020956. 21738596 PMC3126800

[45] Langille MG, Meehan CJ, Koenig JE, Dhanani AS, Rose RA, Howlett SE, Beiko RG 2014 Microbial shifts in the aging mouse gut. Microbiome 2:50. doi:10.1186/s40168-014-0050-9. 25520805 PMC4269096

[46] Baothman OA, Zamzami MA, Taher I, Abubaker J, Abu-Farha M 2016 The role of the gut microbiota in the development of obesity and diabetes. Lipids Health Dis 15:108. doi:10.1186/s12944-016-0278-4. 27317359 PMC4912704

[47] Moeinian M, Ghasemi-Niri SF, Mozaffari S, Abdolghaffari AH, Baeeri M, Navaea-Nigjeh M, Abdollahi M 2014 Beneficial effect of butyrate, Lactobacillus casei and l-carnitine combination in preference to each in experimental colitis. World J Gastroenterol 20:10876–10885. doi:10.3748/wjg.v20.i31.10876. 25152589 PMC4138466

[48] Lin HV, Frassetto A, Kowalik EJ Jr, Nawrocki AR, Lu MM, Kosinski JR, Hubert JA, Szeto D, Yao X, Forrest G, Marsh DJ 2012 Butyrate and propionate protect against diet-induced obesity and regulate gut hormones via free fatty acid receptor 3-independent mechanisms. PLoS One 7:e35240. doi:10.1371/journal.pone.0035240. 22506074 PMC3323649

[49] Armougom F, Henry M, Vialettes B, Raccah D, Raoult D 2009 Monitoring bacterial community of human gut microbiota reveals an increase in Lactobacillus in obese patients and methanogens in anorexic patients. PLoS One 4:e7125. doi:10.1371/journal.pone.0007125. 19774074 PMC2742902

[50] Aronsson L, Huang Y, Parini P, Korach-André M, Håkansson J, Gustafsson J-Å, Pettersson S, Arulampalam V, Rafter J 2010 Decreased fat storage by Lactobacillus paracasei is associated with increased levels of angiopoietin-like 4 protein (ANGPTL4). PLoS One 5:e13087. doi:10.1371/journal.pone.0013087. 20927337 PMC2948012

[51] Million M, Raoult D 2013 Species and strain specificity of *Lactobacillus* probiotics effect on weight regulation. Microb Pathog 55:52–54. doi:10.1016/j.micpath.2012.09.013. 23332210

[52] Ziętak M, Kovatcheva-Datchary P, Markiewicz LH, Ståhlman M, Kozak LP, Bäckhed F 2016 Altered microbiota contributes to reduced diet-induced obesity upon cold exposure. Cell Metab 23:1216–1223. doi:10.1016/j.cmet.2016.05.001. 27304513 PMC4911343

[53] Meehan CJ, Beiko RG 2014 A phylogenomic view of ecological specialization in the Lachnospiraceae, a family of digestive tract-associated bacteria. Genome Biol Evol 6:703–713. doi:10.1093/gbe/evu050. 24625961 PMC3971600

[54] Candon S, Perez-Arroyo A, Marquet C, Valette F, Foray AP, Pelletier B, Milani C, Ventura M, Bach JF, Chatenoud L 2015 Antibiotics in early life alter the gut microbiome and increase disease incidence in a spontaneous mouse model of autoimmune insulin-dependent diabetes. PLoS One 10:e1025448. doi:10.1371/journal.pone.0125448. PMC4430542 25970503

[55] Benito B, Gay-Jordi G, Serrano-Mollar A, Guasch E, Shi Y, Tardif J-C, Brugada J, Nattel S, Mont L 2011 Cardiac arrhythmogenic remodeling in a rat model of long-term intensive exercise training. Circulation 123:13–22. doi:10.1161/CIRCULATIONAHA.110.938282. 21173356

[56] Comeau AM, Li WKW, Tremblay J-É, Carmack EC, Lovejoy C 2011 Arctic Ocean microbial community structure before and after the 2007 record sea ice minimum. PLoS One 6:e27492. doi:10.1371/journal.pone.0027492. 22096583 PMC3212577

[57] Zhang J, Kobert K, Flouri T, Stamatakis A 2014 PEAR: a fast and accurate Illumina Paired-End reAd mergeR. Bioinformatics 30:614–620. doi:10.1093/bioinformatics/btt593. 24142950 PMC3933873

[58] Caporaso JG, Kuczynski J, Stombaugh J, Bittinger K, Bushman FD, Costello EK, Fierer N, Peña AG, Goodrich JK, Gordon JI, Huttley GA, Kelley ST, Knights D, Koenig JE, Ley RE, Lozupone CA, McDonald D, Muegge BD, Pirrung M, Reeder J, Sevinsky JR, Turnbaugh PJ, Walters WA, Widmann J, Yatsunenko T, Zaneveld J, Knight R 2010 QIIME allows analysis of high-throughput community sequencing data. Nat Methods 7:335–336. doi:10.1038/nmeth.f.303. 20383131 PMC3156573

[59] Rideout JR, He Y, Navas-Molina JA, Walters WA, Ursell LK, Gibbons SM, Chase J, McDonald D, Gonzalez A, Robbins-Pianka A, Clemente JC, Gilbert JA, Huse SM, Zhou HW, Knight R, Caporaso JG 2014 Subsampled open-reference clustering creates consistent, comprehensive OTU definitions and scales to billions of sequences. PeerJ 2:e545. doi:10.7717/peerj.545. 25177538 PMC4145071

[60] McDonald D, Price MN, Goodrich J, Nawrocki EP, DeSantis TZ, Probst A, Andersen GL, Knight R, Hugenholtz P 2012 An improved Greengenes taxonomy with explicit ranks for ecological and evolutionary analyses of bacteria and archaea. ISME J 6:610–618. doi:10.1038/ismej.2011.139. 22134646 PMC3280142

[61] Kopylova E, Noé L, Touzet H 2012 SortMeRNA: fast and accurate filtering of ribosomal RNAs in metatranscriptomic data. Bioinformatics 28:3211–3217. doi:10.1093/bioinformatics/bts611. 23071270

[62] Comeau AM, Douglas GM, Langille MGI 2017 Microbiome helper: a custom and streamlined workflow for microbiome research. mSystems 2:e00127-16. doi:10.1128/mSystems.00127-16. 28066818 PMC5209531

[63] Lozupone C, Knight R 2005 UniFrac: a new phylogenetic method for comparing microbial communities. Appl Environ Microbiol 71:8228–8235. doi:10.1128/AEM.71.12.8228-8235.2005. 16332807 PMC1317376

[64] Parks DH, Tyson GW, Hugenholtz P, Beiko RG 2014 STAMP: statistical analysis of taxonomic and functional profiles. Bioinformatics 30:3123–3124. doi:10.1093/bioinformatics/btu494. 25061070 PMC4609014

